# Cerebral oxygenation and hemodynamic changes during ephedrine and phenylephrine administration for transient intraoperative hypotension in patients undergoing major abdominal surgery: a randomized controlled trial

**DOI:** 10.1186/s12871-025-02944-z

**Published:** 2025-02-20

**Authors:** Xueyan Li, Yijun Zheng, Jun Zhang

**Affiliations:** 1 Department of Anaesthesia and Critical Care, Fudan University Shanghai Cancer Center, Shanghai, 200032 PR China; 2 Department of oncology, Shanghai Medical College, Fudan University, Shanghai, 200032 PR China; 3 Department of Intensive Care Unit, Fudan University Shanghai Cancer Center, Shanghai, 200032 P.R. China; 4 Department of Anesthesiology, Fudan University Shanghai Cancer Center, and Shanghai Medical College, Fudan University, 270 Dong An Road, Shanghai, 200032 P.R. China

**Keywords:** Hypotension, Phenylephrine, Ephedrine, Cerebral blood flow velocity, Cardiac output, Cerebral autoregulation

## Abstract

**Background:**

Phenylephrine and ephedrine are frequently used vasopressors for treating intraoperative hypotension. However, their impact on cerebral oxygenation and blood flow remains a subject of debate. This study aims to understand their effects on cerebral oxygen saturation and hemodynamics when used for treatment of intraoperative hypotension.

**Methods:**

The adult patients undergoing major abdominal surgery under general anesthesia were randomly assigned into ephedrine (ED) group or phenylephrine (PE) group. They received an intravenous bolus of either ephedrine or phenylephrine for treating intraoperative transient hypotension. The primary outcome was their effects on regional cerebral oxygen saturation (rScO_2_). The secondary outcomes included cerebral hemodynamics middle cerebral artery velocity (MCAvm), pulsatility index (PI), and resistance index (RI), as well as systemic hemodynamics arterial blood pressure (ABP), heart rate (HR), cardiac output (CO), cardiac index (CI), stroke volume (SV) and stroke volume index (SVI). Additionally, two indices of cerebral autoregulation, mean flow index (Mx_a_) and cerebral oximetry index (CO_X_), were calculated in real-time via ICM + software.

**Results:**

Forty patients were included in this study. The initial results showed ephedrine increased rScO_2_ (*p* < 0.001), while phenylephrine increased Mx_a_ (*p* < 0.02) and CO_X_ (*p* < 0.007), respectively. However, upon further linear-mix model analysis, the effects of both drugs on rScO_2_ (*p* = 0.944), Mx_a_ (*p* = 0.093) and CO_X_ (*p* = 0.084) were found to be non-significant. Compared with the hemodynamic parameters during hypotension, the systolic blood pressure (SBP) (*p* < 0.001), diastolic blood pressure (DBP) (*p* < 0.001), mean arterial pressure (MAP) (*p* < 0.001), and MCAvm (*p* < 0.001) significantly increased after both ephedrine and phenylephrine administration. However, no significant differences were found between the two groups in terms of the changes in MAP (*p* = 0.549) and MCAvm (*p* = 0.173). And there were significant increases in CO (*p* < 0.001), HR (*p* < 0.001), and CI (*p* < 0.001) following ephedrine administration, while decreases in HR (*p* < 0.001), CO (*p* < 0.001), and CI (*p* < 0.001) after phenylephrine administration.

**Conclusion:**

In the management of intraoperative hypotension, both phenylephrine and ephedrine effectively increase MAP and MCAvm, albeit with their differential effects on CO and HR. It seems that neither vasopressor has a significant impact on cerebral oxygenation and cerebral autoregulation.

**Supplementary Information:**

The online version contains supplementary material available at 10.1186/s12871-025-02944-z.

## Introduction

Hypotension is a common occurrence during the perioperative period, primarily due to factors such as blood loss, vasodilation, and inadequate intravascular volume. Persistent hypotension can lead to organ hypoperfusion and damage [1, 2]. For instance, systemic hypotension may cause a substantial reduction in cerebral blood flow (CBF) below a critical threshold [3], potentially resulting in cerebral ischaemia, neuronal death, and brain dysfunction. Cerebral circulation is regulated by a complex and somewhat unpredictable autoregulatory mechanism, the details of which contribute to the variability in how different vasopressors affect both cerebral and systemic circulation.

An intravenous bolus of ephedrine or phenylephrine is a well-established treatment for transient intraoperative hypotension. Several studies have shown that phenylephrine can effectively elevate blood pressure and CBF; however, it may simultaneously decrease cardiac output (CO) and regional cerebral oxygen saturation (rScO_2_) or may have no significant effect on rScO_2_ [4–8]. In contrast, ephedrine has been shown to enhance both CO and rScO_2_ while increasing blood pressure and CBF [9, 10]. Consequently, the hemodynamic effects of these agents may differentially impact the outcomes of patients undergoing surgery. Ma et al. demonstrated that phenylephrine use is associated with an increased risk of postoperative delirium [11]. Therefore, the rational administration of vasopressors for intraoperative hemodynamic management is crucial for optimising postoperative outcomes in surgical patients.

Considering the above background, with the aim of addressing previous controversies regarding the effects of ephedrine and phenylephrine on cerebral oxygenation and haemodynamics during their administration to manage intraoperative transient hypotension, we conducted a randomised, double-blind, controlled clinical trial in adult patients undergoing major abdominal surgery under general anaesthesia. We hypothesised that ephedrine would have a lower influence on cerebral oxygenation and haemodynamics compared to phenylephrine.

## Methods

### Patient population

This single-centre, prospective, randomised controlled study was approved by the Institutional Review Board of Fudan University Shanghai Cancer Centre (FUSCC, approval number: 2211265-7, EC chair: Zhen Chen) and registered in the Chinese Clinical Trial Registry (Identifier: ChiCTR2300069195; Website: https://www.chictr.org.cn/showproj.html?proj=188364; date of registration: March 9, 2023; principal investigator: Jun Zhang). The participants were recruited from March 10, 2023, to November 30, 2023, at FUSCC. Written informed consent was obtained from all participants before enrolment. This study was conducted in accordance with relevant CONSORT guidelines (Supplementary File 1).

Adult patients scheduled for major abdominal surgery under general anaesthesia were recruited. The inclusion criteria were American Society of Anesthesiologists (ASA) physical status I or II and aged between 18 and 64 years. Exclusion criteria were a history of cardiac or neurological surgery, cerebrovascular or cardiac disease, pregnancy, and refusal to participate in the study.

### Anaesthetic management

The patients were required to fast for a minimum of 8 h prior to surgery. Upon arrival at the operating room, standard monitoring was initiated, including electrocardiography, non-invasive blood pressure measurement, and pulse oximetry. Before inducing anaesthesia, an epidural puncture was performed. A radial arterial line was established for continuous invasive arterial blood pressure (ABP) monitoring and blood gas analysis, and a single-lumen central venous catheter was inserted through the right internal jugular vein to facilitate medication administration and fluid infusion.

Anaesthesia was induced by intravenous administration of etomidate 0.3–0.5 mg/kg, sufentanil 0.3–0.5 µg/kg, and rocuronium 0.9 mg/kg to facilitate endotracheal intubation. General anaesthesia was maintained with 1.5–2% sevoflurane in a 50% oxygen mixture, with intermittent rocuronium administration as needed for the maintenance of neuromuscular relaxation. Ventilation settings were adjusted to a tidal volume of 6–8 mL/kg and a respiratory rate between 10 and 12 breaths/min, aiming to maintain the end-tidal carbon dioxide partial pressure (PetCO_2_) within 35–40 mmHg. Epidural anaesthesia was initiated for perioperative analgesia using an indwelling catheter with intermittent injections of 0.375% ropivacaine (volume, 3–5 mL).

### Monitoring of cerebral oxygenation and hemodynamics

#### Monitoring of cerebral oxygenation

Cerebral oxygenation (rScO_2_) was monitored using cerebral oximetry (INVOS 5100 C, Medtronic, USA) through near-infrared spectroscopy (NIRS) technology, which involved placing a cerebral oxygenation monitor on the patient’s forehead, with NIRS optode sensors positioned bilaterally, 1–2 cm superior to the eyebrows. The sensor’s edges were secured with tape to minimise ambient-light interference, thereby ensuring accurate real-time rScO_2_ values. NIRS data from the ipsilateral brain hemisphere corresponding to the side examined using transcranial Doppler (TCD) probe were recorded.

#### Monitoring of cerebral circulation

Cerebral circulation was monitored using a TCD system (EMS-9 PB, Shenzhen Delica Medical Equipment Co., Ltd., China). A 2 MHz hand-held ultrasound probe was positioned over the temporal bone to detect the blood flow of the middle cerebral artery (MCA) on both sides, typically located at an insonation depth ranging from 45 to 56 mm. The side with the clearer blood flow signal was selected, and the optimal site for obtaining the strongest signal was identified by adjusting the angle of the probe. The optimal site was marked, and the ultrasound probe was secured with a headband to maintain the angle for continuous monitoring. Given the high variability between operators, TCD measurements of the MCA, including the mean cerebral blood velocity of the MCA (MCAvm), pulsatility index (PI), and resistance index (RI), were all performed by a single operator (XL).

#### Monitoring of systemic circulation

To monitor systemic circulation, a 20G catheter was inserted into the radial artery and connected to a disposable ProAQT (PV8810) transducer from PulsioFlex (PC4000, PULSION Medical Systems, Germany), which monitors invasive ABP and CO based on pulse wave analysis [12]. Throughout the surgical procedure, haemodynamic parameters, including SV, CO, invasive ABP, and heart rate (HR), were meticulously documented.

### Calculation of derived indices

To assess cerebral autoregulation, two derived indices were calculated in real time. The mean flow index (Mx_a_), which reflects the dynamic correlation between cerebral blood flow velocity (CBFV) and invasive ABP, was continuously monitored using ICM + software (Cambridge Enterprise, Cambridge, UK). The mean cerebral blood velocity (MCAvm) was used as a surrogate marker to assess changes in CBF. Similarly, the cerebral oximetry index (COx) was defined as the dynamic correlation coefficient between brain tissue oxygenation, measured by NIRS, and invasive ABP. Both invasive ABP and rScO_2_ values were converted into digital signals via an analogue-to-digital converter. These signals were captured and recorded in real time using ICM + software, which also facilitated the integration and computation of COx [13].

### Intervention

#### Patient enrolment and randomisation

**Fig. 1:**
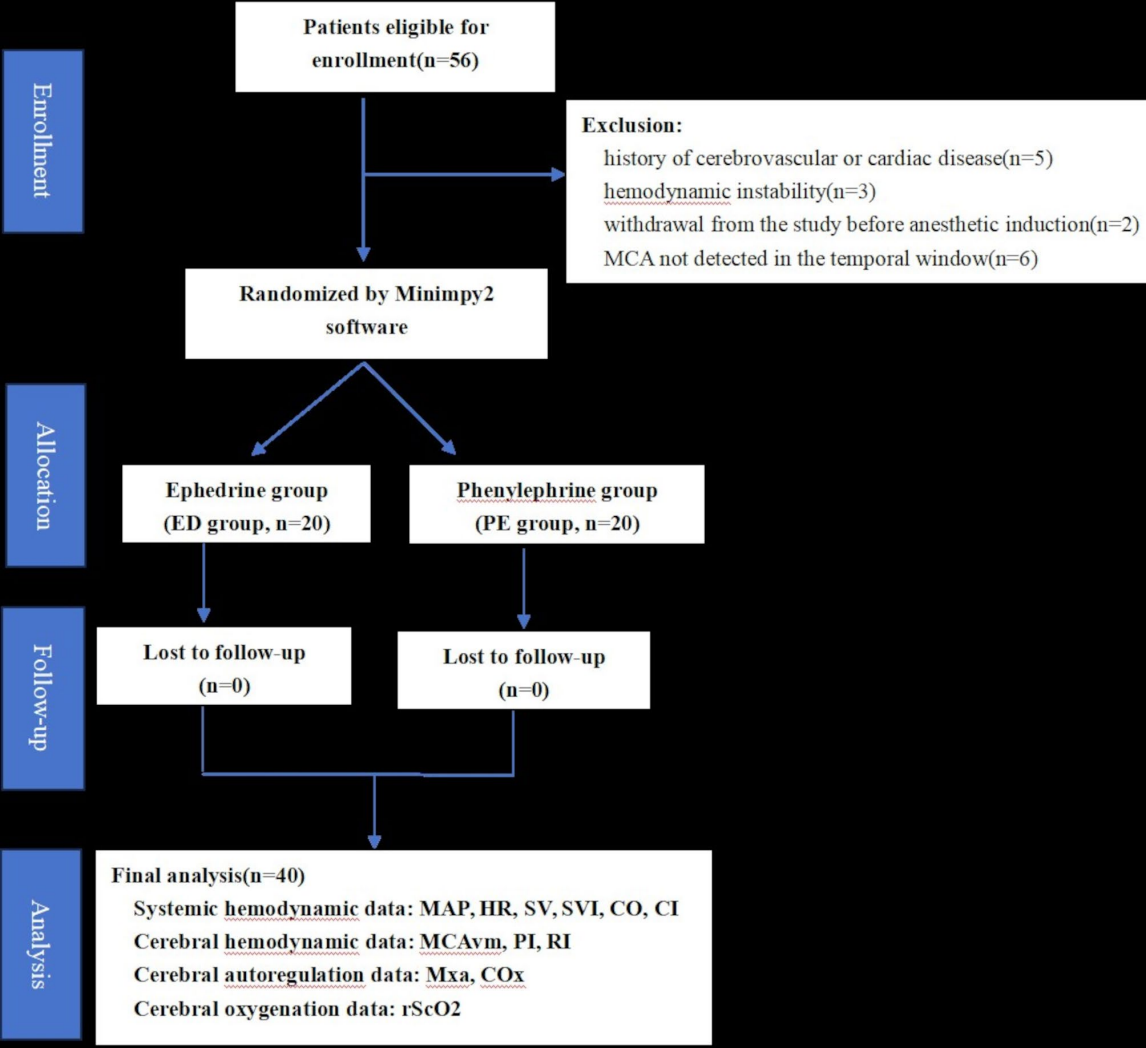
Flowchart of patient enrollment. MAP = Mean arterial pressure; HR = heart rate; SV = stroke volume; SVI = stroke volume index; CO = cardiac output; CI = cardiac index; MCAvm = mean blood velocity of the middle cerebral artery; PI = pulsatility index; RI = resistance index; CO_X_ = cerebral oximetry index; Mx_a_ = mean flow index; rScO_2_ = regional cerebral oxygen saturation

Enrolled patients were randomly allocated to receive either ephedrine (ED group) or phenylephrine (PE group) following a transient hypotension event. Group assignments were performed using opaque sealed envelopes prepared by a research nurse who was not involved in the study. A non-anonymised anaesthesia provider prepared and administered the vasopressors according to the study protocol. In the ED group, patients received a bolus of 1 mL (6 mg/mL) ephedrine intravenously, while in the PE group, patients received a bolus of 1 mL (80 µg/mL) phenylephrine intravenously. The investigator and data analyst were blinded to the group allocation. Refractory hypotension events, such as massive bleeding and anaphylactic shock, were excluded from the analysis; thus, only data from patients in a state of “transient hypotension” were collected. A flowchart of patient enrolment is shown in Fig. 1.

As a commonly accepted threshold [14], hypotension was defined as a mean arterial pressure (MAP) less than 60 mmHg or systolic blood pressure (SBP) less than 90 mmHg. While a corrected hypotension was defined as a MAP ≥ 60 mmHg or SBP ≥ 90 mmHg.

#### Data collection

**Fig. 2:**
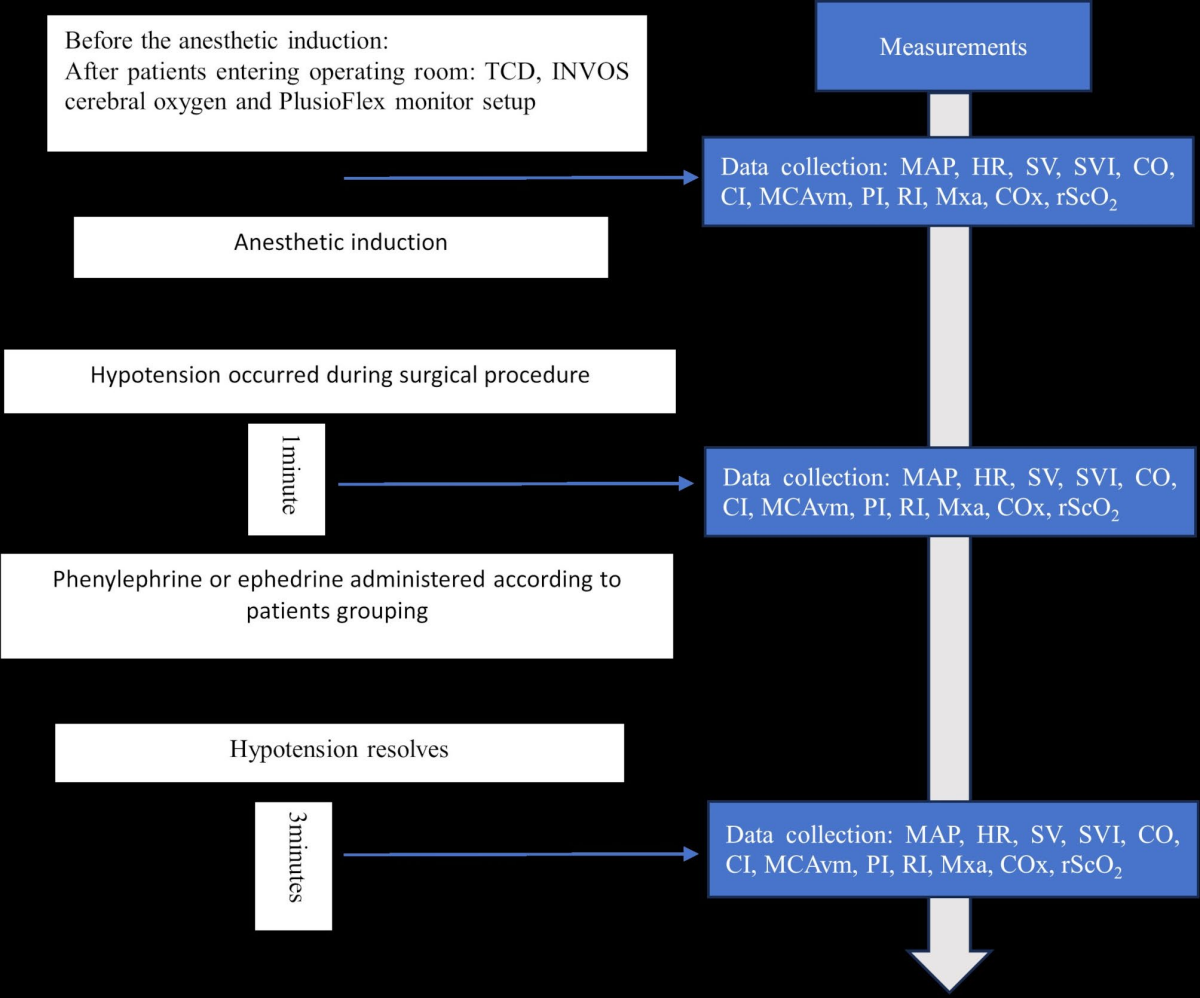
The study protocol and data collection. SBP = systolic blood pressure, MAP = Mean arterial pressure, DBP = diastolic blood pressure, HR = heart rate, MCAvm = mean blood velocity of the middle cerebral artery, rScO_2_ = regional cerebral oxygen saturation, SV = stroke volume, SVI = stroke volume index, CO = cardiac output, CI = cardiac index, CO_X_ = cerebral oximetry index, PI = pulsatility index, RI = resistance index, Mx_a_ = mean flow index

To eliminate potential baseline differences between the two groups, cerebral haemodynamic variables, including rScO_2_, MCAvm, PI, RI, COx, and Mx_a_, as well as systemic haemodynamic variables, including SV, CO, MAP, and HR, were documented before anaesthetic induction. (FiO_2_ = 0.21) and 5 min after anaesthetic induction (FiO_2_ = 1.0) as baseline data. These indicators were recorded when hypotension occurred intraoperatively for at least 1 min (FiO_2_ = 0.5). A bolus of ephedrine or phenylephrine was administered intravenously (IV). These data were documented once again after hypotension resolved and remained stable for at least 3–5 min for accurately calculating Mx_a_ and COx (Fig. 2). The average values from three recordings during hypotension and after blood pressure elevation were used for analysis. If another hypotension event occurred, treatment was repeated as described above.

The demographic and clinical data of the patients in the two groups were collected using electronic health and anaesthesia systems.

### Study endpoints

#### Primary outcome

The primary outcome was the effect of ephedrine and phenylephrine on rScO_2_.

#### Secondary outcomes

Changes in cerebral haemodynamic parameters (MCAvm, PI, and RI) before and after vasopressor administration and changes in systemic haemodynamic parameters, including CO, CI, SV, SVI, MAP, and HR, were assessed. The analysis also included two indices of cerebral autoregulation, Mx_a_ and CO_X_, during the episodes of hypotension. Furthermore, the correlations between these changes in cerebral and systemic haemodynamics were examined.

### Statistical analysis

The sample size was determined based on the requirement to detect a 5% decrease rScO_2_ caused by phenylephrine administration, as reported previously [15]. To achieve a statistical power of 0.8 with a significance level of ɑ=0.05, a minimum of 18 patients were required per group, as determined using the PASS 15.0 software (NCSS, USA). Anticipating a dropout rate of 10% throughout the study, a total of 40 patients were planned for recruitment.

Categorical variables were presented as numbers and percentages. Continuous variables are expressed as mean (SD) or median (IQR), depending on their distribution. Data were compared using paired and unpaired Student’s t-tests, Wilcoxon rank-sum tests, or Fisher’s exact tests, as appropriate, using IBM SPSS Statistics version 25 (IBM Corp., Armonk, NY, USA). Because this two-treatment study involved repeated measurements, the time course of haemodynamic variables before and after vasopressor therapy was compared between groups by a linear-mixed model using R (version 4.4.2; R Foundation for Statistical Computing, Vienna, Austria), as described by Meng et al. [15]. The relationships between changes in systemic and cerebral haemodynamic variables, such as MAP, MCAvm, CO, and MCAvm, during the study period were assessed using the Spearman correlation analysis. Statistical significance was set at *p* < 0.05. significance.

## Results

Fifty-six patients were enrolled in this study. Sixteen patients were excluded from the study for several reasons. Consequently, 40 patients were included in the final analysis as depicted in Fig. 1. Hypotension episodes were recorded in the PE and ED group experiencing 86 and 75 episodes, respectively.

### Patients’ characteristics and baseline data

The demographic and clinical profiles of the patients in the ED (*n* = 20) and PE (*n* = 20) groups were comparable, as listed in Table 1. The peri-induction systemic and cerebral haemodynamic parameters, as well as rScO_2_ are listed in Supplementary Table 1. There were no differences before or after inducing anaesthesia between the two groups.

**Table 1:** The demographic data and clinical characteristics

	Phenylephrine group (*n* = 20)	Ephedrine group (*n* = 20)	*p* value
Gender (male/female)	11/9	9/11	0.527
Age, years	49.5(8.8)	52.6(9.0)	0.276
Height, cm	168.1(6.3)	165.3(6.7)	0.179
Weight, kg	64.6(9.7)	61.7(9.7)	0.851
BMI	22.8(2.9)	22.5(2.6)	0.716
ASA physical status (I/II)	3/17	4/16	0.677
Amount of infused fluid (ml)	1478.2(107.7)	1499.6(223.3)	0.773
Blood loss (ml)	269.6(15.8)	291.5(13.1)	0.651
Duration of surgery (min)	312.2(46.3)	308.7(58.6)	0.883
Duration of anesthesia (min)	344.5(27.8)	340.0(32.6)	0.896
Smokers/non-smokers	9.0/11.0	10.0/10.0	0.752

Note: Data are shown as mean (SD) or number. BMI: body mass index; ASA: American Society of Anesthesiology

### Oxygenation and hemodynamic responses to vasopressors administration

In the PE group, there was no significant change in rScO_2_ levels (*p* = 0.886) after phenylephrine administration. The results also revealed a significant decrease in HR (*p* < 0.001), SV (*p* = 0.002), SVI (*p* = 0.003), CO (*p* < 0.001), and CI (*p* < 0.001), and increases in MAP (*p* < 0.001), MCAvm (*p* < 0.001), Mx_a_ (*p* = 0.020), and Cox (*p* = 0.007). Notably, PI (*p* = 0.061) and RI (*p* = 0.067) remained unchanged from pre-to post-phenylephrine administration (Table 2).

**Table 2:** The systemic and cerebral hemodynamics before and after phenylephrine use (*n* = 86 episodes of hypotension)

Parameters	Before phenylephrine use	After phenylephrine use	Mean difference (95% CI)	*p* value
SBP (mmHg)	88.4(4.3)	107.3(9.1)	18.9(17.0–20.9)	< 0.001
DBP (mmHg)	43.3(5.7)	55.9(8.7)	12.5(11.1–14.0)	< 0.001
MAP (mmHg)	58.4(4.7)	73.1(8.4)	14.7(16.3–18.2)	< 0.001
HR (bpm)	61.3(7.7)	56.2(6.3)	-5.2(-6.2 - -4.3)	< 0001
SV (ml)	84.2(14.4)	80.2(13.9)	-4.0(-6.4 - -1.5)	0.002
SVI (ml/m^2^)	46.8(7.1)	44.7(7.1)	-2.2(-3.6 - -0.8)	0.003
CO (L/min)	5.1(1.2)	4.6(1.1)	-0.6(-0.7 - -0.4)	< 0.001
CI (L/min*m^2^)	2.8(0.5)	2.5(0.5)	-0.3(-0.4 - -0.2)	< 0.001
rScO_2_ (%)	62.3(12.1)	62.2(12.1)	-0.0(-0.5–0.5)	0.886
MCAvm (cm/s)	47.4(14.4)	50.7(13.8)	3.4(2.3–4.5)	< 0.001
PI	1.0[0.9, 1.2]	0.9[0.8, 1.1]	-0.1(-0.1 - -0.0)	0.061
RI	0.6[0.6, 0.7]	0.6[0.6, 0.7]	-0.0(-0.1–0.0)	0.067
CO_X_	0.2[-0.3, 0.5]	0.3[0.1, 0.6]	0.1(0.0–0.2)	0.007
M_Xa_	0.4[0.2, 0.7]	0.5[0.3, 0.7]	0.1(0.0–0.1)	0.020

Note: Data are shown as mean (SD), mean (95% confidence interval (CI)) or median [quartile range] and median (95% CI). ^*^p < 0.05 between two groups (before vs. after, paired Student’s t-test). SBP: systolic blood pressure; MAP: Mean arterial blood pressure; DBP: diastolic blood pressure; HR: heart rate; SV: stroke volume; SVI: stroke volume index; CO: cardiac output; CI: cardiac index; rScO_2_: regional cerebral oxygen saturation; MCAvm: mean blood velocity of the middle cerebral artery; PI: pulsatility index; RI: resistance index; CO_X_: cerebral oximetry index; M_Xa_: mean flow index

In contrast, in the ED group, a significant increase in rScO_2_ (*p* < 0.001) was observed after ephedrine administration. Additionally, MAP (*p* < 0.001), HR (*p* < 0.001), CO (*p* < 0.001), CI (*p* < 0.001) and MCAvm (*p* < 0.001) all significantly increased after ephedrine administration, while SV (*p* = 0.802), SVI (*p* = 0.853), Mx_a_ (*p* = 0.094), COx (*p* = 0.874), PI (*p* = 0.064) and RI (*p* = 0.089) remained unchanged (Table 3).

**Table 3:** The systemic and cerebral hemodynamics before and after ephedrine use (*n* = 77 episodes of hypotension)

Parameters	Before ephedrine use	After ephedrine use	Mean/Median difference (95% CI)	*p* value
SBP (mmHg)	89.9(8.2)	112.2(11.8)	22.3(19.7–24.8)	< 0.001
DBP (mmHg)	45.9(6.7)	55.6(8.1)	9.8(8.4–11.2)	< 0.001
MAP (mmHg)	59.0(6.4)	74.4(8.5)	13.9(12.2–15.7)	< 0.001
HR (bpm)	65.3(10.3)	71.5(12.0)	6.2(5.0–7.4)	< 0.001
SV (ml)	75.5(17.2)	75.8(18.3)	0.3(-2.0–2.6)	0.802
SVI (ml/m^2^)	43.1(9.3)	43.3(9.7)	0.1(-1.2–1.4)	0.853
CO (L/min)	4.9(1.1)	5.3(1.2)	0.4(0.3–0.6)	< 0.001
CI (L/min*m^2^)	2.8(0.7)	3.0(0.7)	0.2(0.2–0.3)	< 0.001
rScO_2_ (%)	61.2(10.5)	62.2(10.0)	1.0(0.5–1.4)	< 0.001
MCAvm (cm/s)	43.6(11.8)	48.9(12.9)	5.3(4.2–6.3)	< 0.001
PI	1.1[0.8, 1.2]	1.0[0.8, 1.2]	-0.1(-0.2–0.2)	0.064
RI	0.6[0.5, 0.6]	0.6[0.5, 0.6]	-0.0(-0.0–0.0)	0.089
CO_X_	0.2[-0.2, 0.7]	0.3[-0.3, 0.6]	0.1(-0.2–0.3)	0.874
M_Xa_	0.4[0.0, 0.7]	0.4[0.2, 0.7]	0.1(-0.0–0.2)	0.094

Note: Data are shown as mean (SD), mean (95% confidence interval (CI)) or median [quartile range] and median (95% CI). ^*^p < 0.05 between two groups (before vs. after, paired Student’s t-test). SBP: systolic blood pressure; MAP: Mean arterial blood pressure; DBP: diastolic blood pressure; HR: heart rate; SV: stroke volume; SVI: stroke volume index; CO: cardiac output; CI: cardiac index; rScO_2_: regional cerebral oxygen saturation; MCAvm: mean blood velocity of the middle cerebral artery; PI: pulsatility index; RI: resistance index; CO_X_: cerebral oximetry index; M_Xa_: mean flow index

**Fig. 3:**
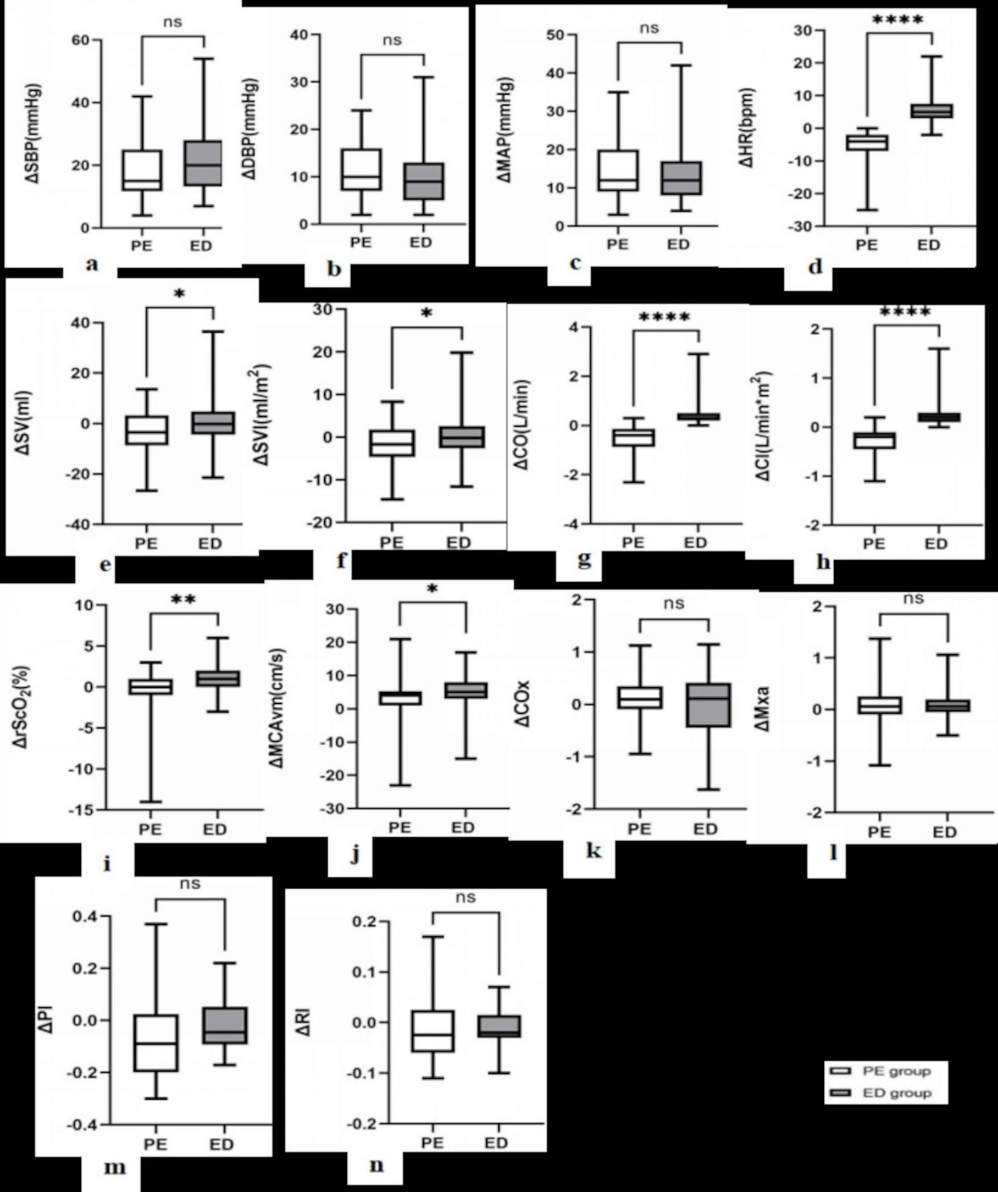
The comparisons between changes in hemodynamics before and after vasopressors administration. The data are presented with differences in mean values (SD, 95% confidence interval (CI)) or median (95% CI). (**a**) difference in systolic pressure (ΔSBP), PE group: 19.0(9.2) mmHg, 95% CI: 17.0–20.9 mmHg vs. ED group: 21.7(9.7) mmHg, 95% CI: 19.4–24.0 mmHg, *p* = 0.070; (**b**) difference in diastolic pressure (ΔDBP), PE group: 11.2(5.2) mmHg, 95% CI: 10.0–6.1 mmHg vs. ED group: 10.0(6.1) mmHg, 95% CI: 8.4–11.2 mmHg, *p* = 0.132; (**c**) difference in mean arterial pressure (ΔMAP), PE group: 14.7(7.4) mmHg, 95% CI 13.2–16.3 mmHg vs. ED group: 14.0(7.4) mmHg, 95%CI: 12.2–15.7 mmHg, *p* = 0.492; (**d**) difference in heart rate (ΔHR), PE group: -5.1(4.3) bpm, 95% CI:-6.1 - -4.2 bpm vs. ED group: 5.9(4.8) beats per minute (bpm), 95% CI: 4.8–7.1 bpm, *p* < 0.0001; (**e**) difference in stroke volume (ΔSV), PE group: -4.0(8.9) ml, 95% CI:-6.4 - -1.5 ml vs. ED group: 0.3(8.5) ml, 95% CI:-2.0–2.6 ml, *p* = 0.013; (**f**) difference in stroke volume index (ΔSVI), PE group: -2.2(5.1) ml/m2, 95% CI:-3.6 - -6.8 ml/m^2^ vs. ED group: 0.1(4.8) ml/m^2^, 95% CI:-1.2–1.4ml/m^2^, *p* = 0.018; (**g**) difference in cardiac output (ΔCO), PE group: -0.6(0.6) L/min, 95% CI: -0.7 - -0.4 L/min vs. ED group: 0.4(0.5) L/min, 95% CI:0.3–0.6 L/min, *p* < 0.0001; (**h**) difference in cardiac index (ΔCI), PE group: -0.3(0.3) L/min*m^2^, 95% CI: -0.4 - -0.2 L/min*m^2^ vs. ED group: 0.2(0.3) L/min*m^2^, 95% CI: 0.2–0.3 L/min*m^2^, *p* < 0.001; (**i**) difference in regional cerebral oxygenation saturation (ΔrScO_2_), PE group: 0.0(2.3)%, 95% CI: -0.5–0.4% vs. ED group: 1.0(1.9)%, 95% CI:0.5–1.4%, *p* = 0.003; (**j**) difference in mean blood velocity of the middle cerebral artery (ΔMCAvm), PE group: 3.4(5.2) cm/s, 95% CI:2.3–4.5 cm/s vs. ED group: 5.3(4.6) cm/s, 95% CI: 4.2–6.4 cm/s, *p* = 0.016; (**k**) difference in cerebral oximetry index (ΔCOx), PE group: 0.1(0.0- 0.2) vs. ED group: 0.1(-0.1- 0.2), *p* = 0.341; (**l**) difference in mean flow index (ΔMxa), PE group: 0.1(-0.1–0.2) vs. ED group: 0.0(-0.1–0.2), *p* = 0.982; (**m**) difference in pulsatility index (ΔPI), PE group: -0.1(-0.2–0.0) vs. ED group: -0.1(-0.1–0.0), *p* = 0.063 (**n**) difference in resistance index (ΔRI), PE group: -0.0(-0.1–0.0) vs. ED group: -0.0(-0.0–0.0), *p* = 0.504; ns, *p* > 0.05, * *p* ≤ 0.05, ** *p* < 0.001, *** *p* < 0.001, **** *p* < 0.0001

When comparing the changes before and after vasopressor administration at the group level, the ED group exhibited greater changes in HR (ΔHR, *p* < 0.001) (Fig. 3d), SV (ΔSV, *p* = 0.013) (Fig. 3e), SVI (ΔSVI, *p* = 0.018) (Fig. 3f), CO (ΔCO, *p* < 0.001) (Fig. 3g) and CI (ΔCI, *p* < 0.001) (Fig. 3h) than PE group. Further, ephedrine also demonstrated a more pronounced increase in MCAvm (ΔMCAvm, *p* = 0.016) when raising MBP to the same level as phenylephrine did (Fig. 3j). Although the rScO_2_ values were similar after treatments between the two groups [62(12) % vs. 62(10) %, *p* = 0.717], the difference in rScO_2_ (ΔrScO_2_) in ED group was higher than PE group (*p* = 0.003) (Fig. 3i).

Furthermore, a linear-mixed model analysis confirmed the effects of ephedrine and phenylephrine on MAP, HR, CO, CI, PI, RI, and MCAvm. In this case, neither phenylephrine nor ephedrine had a significant effect on rScO_2_ (*P* = 0.944), and the time*group interaction showed no statistically significant difference between the two groups (*p* = 0.167). Additionally, the time*group interaction showed no statistically significant differences between the two groups in terms of MCAvm (*p* = 0.173), SV(*p* = 0.183), and SVI(*p* = 0.199). Moreover, phenylephrine was also found not to change Mx_a_ (*p* = 0.093) and CO_X_ (*p* = 0.084) (supplementary Table 2).

**Fig. 4:**
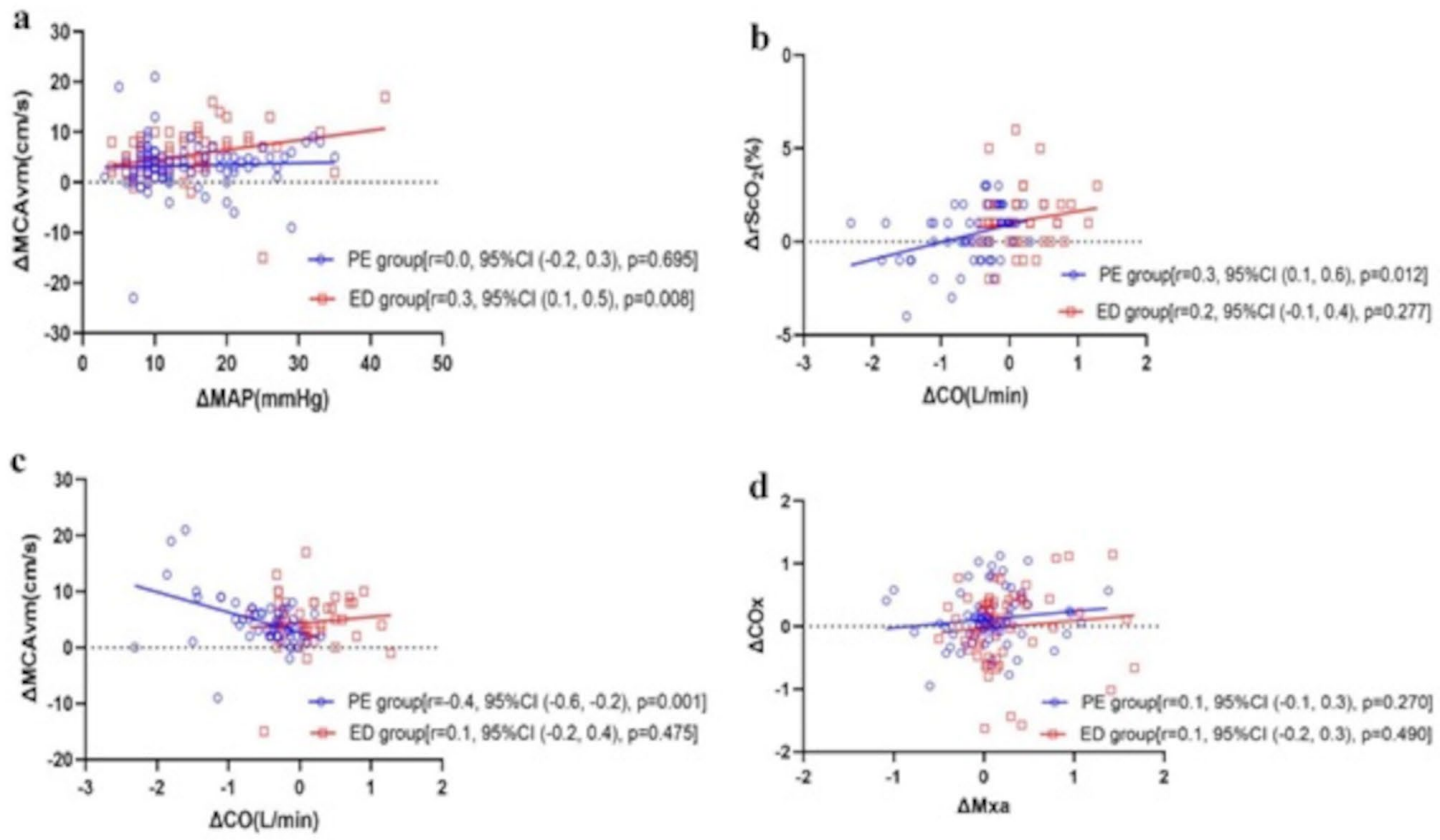
The correlation of changes in hemodynamics after vasopressors administration. (**a**) The correlation between difference of mean arterial pressure (ΔMAP) and difference of mean blood velocity of the middle cerebral artery (ΔMCAvm); (**b**) The correlation between difference of cardiac output (ΔCO) and difference of cerebral oxygenation saturation (ΔScO_2_); (**c**) The correlation between difference of ΔScO_2_ and difference of ΔMCAvm; (**d**) The correlation between difference of mean flow index (ΔMx_a_) and difference of cerebral oximetry index (ΔCOx). Pearson correlation coefficients (r), 95% CI and significance in two groups are shown on the graph

We also analysed the effects of systemic haemodynamic changes on cerebral haemodynamics and oxygenation. In the ED group, changes in MAP positively correlated with changes in MCAvm (Fig. 4a), suggesting that the greater the MAP increase caused by ephedrine treatment, the greater the increase in CBF. In the PE group, changes in CO were negatively correlated with changes in MCAvm but positively correlated with changes in rScO_2_ (Fig. 4b and c), suggesting that as CO decreased with phenylephrine treatment, brain oxygenation decreased, but MCAvm increased, despite an increase in blood pressure. There was no significant correlation between changes in the Mx_a_ and COx in either group (Fig. 4d).

## Discussion

This study investigated the effects of two commonly used vasopressors, ephedrine and phenylephrine, on both cerebral oxygenation and circulation in patients undergoing major abdominal surgery. It also incorporated two dynamic cerebral autoregulation indices, Mx_a_ and COx, to assess their impact on cerebral autoregulation. Our results indicated that ephedrine and phenylephrine differentially influenced systemic haemodynamics but had similar, non-significant effects on cerebral oxygenation, cerebral haemodynamics, and cerebral autoregulation when used to treat intraoperative hypotension. These findings provide valuable insights into the therapeutic choice of vasopressors for perioperative hypotension.

Phenylephrine and ephedrine can both be administered as intravenous boluses to treat transient hypotension. However, phenylephrine acts exclusively on α1 receptors to constrict peripheral blood vessels, whereas ephedrine’s effects on the cardiovascular system, including vasoconstriction and cardiac stimulation, are primarily due to its action on both α1 and β1 adrenergic receptors, with both direct and indirect effects. Consequently, a decrease in CO and HR following phenylephrine administration, and an increase in CO and HR following ephedrine administration, can be readily predicted. Nevertheless, neither phenylephrine nor ephedrine significantly affected SV, suggesting that ephedrine increases CO primarily by increasing HR, while phenylephrine maintains a stable SV by increasing preload and afterload through venous and arterial constriction.

When the tone of the cerebral blood vessels remains stable, changes in TCD-measured blood flow velocity are widely recognised as reliable indicators of CBF changes. Given their little impact on cerebral vascular tone due to the sporadic presence of α1 receptors in the cerebral vasculature, as well as the stable PetCO_2_ levels during surgery, the effects of phenylephrine and ephedrine on CBF can be investigated through measuring MCAvm. The PI and RI did not significantly increase after phenylephrine administration, implying that the increase in MCAvm was not attributable to cerebral vasoconstriction. Both ephedrine and phenylephrine increased MCAvm with a similar increase in MAP levels, which is consistent with previous studies [6, 16, 17]. Therefore, our results indicate that, although they differentially influence CO, both vasopressors similarly increase CBF, as evidenced by the elevated MCAvm.

Although increased blood pressure is expected to improve cerebral oxygenation, vasopressor use may impair cerebral oxygenation during general anaesthesia [18]. However, the effects of phenylephrine and ephedrine on rScO_2_ remain debated. Several studies suggest that phenylephrine administration decreases rScO_2_ by 3 to 20%, whereas ephedrine treatment maintains or increases rScO_2_ [4, 15, 19, 20]. The mechanism for this reduction in rScO_2_ may be related to a decrease in CO induced by phenylephrine or the extracranial vasoconstrictive effect of phenylephrine, known as extracranial contamination [21], as α1-receptors are sporadically present in the brain vasculature but are abundantly present in peripheral vasculature [22]. However, the effects of vasopressors on rScO_2_ vary depending on methodologies and paradigms of vasopressor use. For instance, near-infrared time-resolved spectroscopy measurement, a method with minimal extracranial contamination, indicates that phenylephrine has little impact on rScO_2_ during laparoscopic surgery for treating hypotension [23]. Additionally, the changes in rScO_2_ in response to the use of ephedrine and phenylephrine were found to be very similar and clinically insignificant, even though MAP was sufficiently increased by both. Furthermore, a positron emission tomography (PET) study, in which vasopressors were administered as continuous infusions, found no significant change in rScO_2_ in normal brain regions of patients with brain tumours; however, ephedrine significantly increased CBF and rScO_2_ [24]. These findings align with our results, showing that phenylephrine did not reduce rScO_2_, even when measured using NIRS, as in previous studies [4, 15, 25]. A possible explanation is that extracranial contamination may have been minimised in our study due to the wide headband tightly securing the ultrasound probe around the forehead [26]. Compared to phenylephrine, ephedrine had a statistically higher but clinically insignificant increase in rScO_2_ (+ 1%) in our study using paired *t*-test. By using a linear-mixed model analysis, we found that ephedrine no longer increased rScO_2_, suggesting the significance in the result from the paired *t*-test could be caused by random effects since the study involved repeated measurements. The rScO_2_ values depend on the balance between cerebral oxygen supply and consumption, which remains stable during the intraoperative period. Given that cerebral haemoglobin, PaCO_2_, and PaO_2_ were stable before and after phenylephrine and ephedrine use, the stability of rScO_2_ values in our study suggests that this balance is preserved in a putatively healthy brain following vasopressor administration for intraoperative transient hypotension.

Cerebral autoregulation actively ensures that CBF remains constant over a range of MAP (50–150 mmHg) by balancing the constriction and dilation of the cerebral vasculature. Impaired cerebral autoregulation in elderly and high-risk patients undergoing major non-cardiac surgery is associated with failure of cognitive recovery in the early postoperative period, as well as 1-month mortality and morbidity [27]. Recently, a multicentre retrospective cohort study demonstrated that the administration of phenylephrine, compared to ephedrine, for hypotension management during general anaesthesia was associated with a higher risk of postoperative delirium within 7 days in adults undergoing general anaesthesia for non-cardiac, non-neurosurgical procedures [11]. This could be attributed to impaired cerebral autoregulation or cerebral perfusion due to vasoconstriction induced by phenylephrine treatment. However, in another prospective cohort study, impairment of intraoperative cerebral autoregulation did not predict early postoperative cognitive dysfunction (POCD) in elderly patients undergoing major non-cardiac surgery [28]. In the present study, two indices, Mxa and COx, were used to estimate dynamic cerebral autoregulation using non-invasive methods. We assumed that cerebral autoregulation was intact before surgery in our patients. Mxa in healthy volunteers is reported to be around 0.40 [29]; however, in patients with traumatic brain injury, the most commonly used Mxa threshold for preserved versus impaired cerebral autoregulation is 0.3, showing low to moderate accuracy [30, 31]. COx is sensitive in detecting impaired cerebral autoregulation due to hypotension when it is above a threshold of 0.36 [32, 33]. Our results showed that phenylephrine, rather than ephedrine, elevated Mx_a_ and COx, However, upon switching to a linear-mixed model analysis, neither phenylephrine nor ephedrine had a significant impact on Mx_a_ or COx. This implies that phenylephrine and ephedrine did not differentially affect CBF or cerebral autoregulation in our study, which is consistent with the results of previous studies [19, 34]. This is likely attributable to the younger patients enrolled in our study and their blood pressure remaining within the autoregulated range.

The present study has several limitations. First, this clinical trial had a small sample size, and only middle-aged surgical patients without significant cerebrovascular disease were included. Therefore, the findings of this study may not be applicable to elderly or paediatric patients, or those with preexisting vascular pathologies. Secondly, for patient safety, the duration of hypotension was intentionally kept brief (not reaching the ideal duration of five minutes), which may have resulted in lower temporal resolution for the calculation of Mxa and COx. Third, we did not consider the smoking history of the patients, which could potentially affect vascular tone. Future studies will evaluate the correlation between COx and Mx_a_ by incorporating additional techniques and assessment parameters to provide a more comprehensive understanding of the effects of vasopressors on cerebral autoregulation.

## Conclusion

This randomised controlled clinical trial revealed that, in the management of intraoperative transient hypotension, both phenylephrine and ephedrine exert comparable effects on cerebral oxygenation and haemodynamics. Specifically, an intravenous bolus of phenylephrine increased CBF while simultaneously decreasing CO and HR. In contrast, ephedrine elevated CBF, CO, and HR. Notably, neither vasopressor appeared to significantly affect rScO_2_ nor impair cerebral autoregulation.

## Electronic supplementary material

Below is the link to the electronic supplementary material.

Supplementary Material 1

Supplementary Material 2

## Data Availability

No datasets were generated or analysed during the current study.

## Declarations

### Declaration of the use of Generative AI in writing

The authors declare that they have not used generative AI in writing.

## Abbreviations

SBP: Systolic blood pressure
MAP: Mean arterial pressure
DBP: Diastolic blood pressure
ABP: Arterial blood pressure
HR: Heart rate
CBF: Cerebral blood flow
MCAvm: Mean blood velocity of the middle cerebral artery
rScO_2_: Regional cerebral oxygen saturation
SV: Stroke volume
SVI: Stroke volume index
CO: Cardiac output
CI: Cardiac index
CO_X_: Cerebral oximetry index
PI: Pulsatility index
RI: Resistance index
Mx_a_: Mean flow index
TCD: Transcranial doppler
NIRS: The near-infrared spectroscopy
ASA: American Society of Anesthesiologists
P_ET_CO_2_: End-tidal carbon dioxide partial pressure
