# Supplementary material for: Cerebral oxygenation and hemodynamic changes during ephedrine and phenylephrine administration for transient intraoperative hypotension in patients undergoing major abdominal surgery: a randomized controlled trial
Source: BMC Anesthesiol. 2025 Feb 20;25:87. doi: 10.1186/s12871-025-02944-z (PMC11841356; doi:10.1186/s12871-025-02944-z)
Supplement: Supplementary file 2 — Supplementary Material 2 [file 12871_2025_2944_MOESM2_ESM.docx]

**Supplementary Table 1.** Baseline systemic and cerebral hemodynamics data before and after anaesthetic induction

|  | | Phenylephrine group  (n=20) | Ephedrine group  (n=20) | | *p* value |
| --- | --- | --- | --- | --- | --- |
| *Pre-induction* (FiO_2_=0.21) |  | | |  | |
| SBP (mmHg) | | 147.0(23.0) | 146.0(21.0) | | 0.875 |
| DBP (mmHg) | | 74.0(11.0) | 69.0(13.0) | | 0.220 |
| MAP (mmHg) | | 98.0(14.0) | 95.0(15.0) | | 0.425 |
| HR (bpm) | | 73.0(12.0) | 70.0(9.0) | | 0.300 |
| Hb (g/dl) | | 13.9(1.6) | 14.5(2.8) | | 0.518 |
| CO (L/min) | | 6.1(2.0) | 5.7(1.0) | | 0.450 |
| CI (L/min.m^2^) | | 3.3(0.8) | 3.2(0.5) | | 0.810 |
| SV(ml) | | 83.7(21.7) | 88.1(23.6) | | 0.538 |
| SVI(ml/m^2^) | | 46.4(12.4) | 50.2(13.4) | | 0.352 |
| PaO2 (mmHg) | | 78.0(12.0) | 75.0(11.0) | | 0.361 |
| rScO2 (%) | | 64.0 (10.0) | 59.0(10.0) | | 0.181 |
| MCAvm (cm/s) | | 57.0(10.0) | 63.0(9.0) | | 0.113 |
| PI | | 1.3(0.4) | 1.4(0.5) | | 0.782 |
| RI | | 0.7(0.1) | 0.7(0.1) | | 0.881 |
| COx | | 0.2[-0.1, 0.3] | 0.1[-0.2, 0.4] | | 0.883 |
| Mxa | | 0.3[0.2, 0.4] | 0.2[0.1, 0.4] | | 0.231 |
| *Post-induction* (FiO_2_=1.0) | | | | | |
| SBP (mmHg) | | 119.0(18.0) | 111.0(11.0) | | 0.120 |
| DBP (mmHg) | | 59.0(12.0) | 55.0(10.0) | | 0.252 |
| MAP (mmHg) | | 79.0(13.0) | 72.0(9.0) | | 0.056 |
| HR (bpm) | | 71.0(10.0) | 69.0(7.0) | | 0.680 |
| Hb (g/dl) | | 12.3(1.5) | 12.8(1.9) | | 0.920 |
| CO (L/min) | | 4.8(1.6) | 4.6(0.7) | | 0.701 |
| CI (L/min*m^2^) | | 2.6(0.8) | 2.6(0.4) | | 0.912 |
| SV(ml) | | 69.4(19.1) | 78.1(23.3) | | 0.205 |
| SVI(ml/m^2^) | | 38.6(11.2) | 44.5(13.4) | | 0.141 |
| PaO2 (mmHg) | | 375.0(15.0) | 360.0(13.0) | | 0.672 |
| rScO2 (%) | | 67.0(9.0) | 65.0(9.0) | | 0.500 |
| MCAvm (cm/s) | | 44.0(4.0) | 43.0(9.0) | | 0.753 |
| PI | | 1.1(0.3) | 1.2(0.3) | | 0.670 |
| RI | | 0.6(0.1) | 0.7(0.1) | | 0.730 |
| COx | | 0.2[-0.1, 0.3] | 0.2[-0.3, 0.3] | | 0.640 |
| Mxa | | 0.4[0.2, 0.5] | 0.3[0.1, 0.4] | | 0.192 |

**Note:** Data are shown as mean (SD) or median [quartile range]. ^*^p<0.05 between two groups. SBP: systolic blood pressure; MAP: Mean arterial blood pressure; DBP: diastolic blood pressure; HR: heart rate; SV: stroke volume; SVI: stroke volume index; CO: cardiac output; CI: cardiac index; rScO_2_: regional cerebral oxygen saturation; MCAvm: mean blood velocity of the middle cerebral artery_;_ PI: pulsatility index_;_ RI: resistance index; CO_X_: cerebral oximetry index; M_Xa_: mean flow index.

**Supplementary Table 2.** The change in systemic and cerebral hemodynamics before and after phenylephrine or ephedrine use with a linear-mixed model analysis

| **Parameters** | **Group** | | **Time** | | **Group*Time** | |
| --- | --- | --- | --- | --- | --- | --- |
|  | **β (95% CI)** | ***p* value** | **β (95% CI)** | ***p* value** | **β (95% CI)** | ***p* value** |
| SBP (mmHg) | 1.9(-1.8,5.7) | 0.318 | 18.9(16.7,21.2) | <0.001* | 3.3(0.0,6.6) | 0.049* |
| DBP (mmHg) | 1.4(-2.5,5.2) | 0.486 | 12.5(10.9,14.1) | <0.001* | -2.8(-5.1,-0.4) | 0.024* |
| MAP (mmHg) | 1.7(-1.7,5.0) | 0.332 | 14.7(13.0,16.4) | <0.001* | -0.8(-3.3,1.8) | 0.549 |
| HR (bpm) | 3.1(-1.9,8.1) | 0.231 | -5.2(-7.0,-3.5) | <0.001* | 11.4(8.9,14.0) | <0.001* |
| SV (ml) | -10.2(-22.8,2.4) | 0.131 | -4.0(-8.4,0.5) | 0.082 | 4.3(-2.0,10.5) | 0.183 |
| SVI (ml/m^2^) | -2.9(-9.1,3.3) | 0.376 | -2.2(-4.7,0.3) | 0.087 | 2.3(-1.2,5.8) | 0.199 |
| CO (L/min) | -0.3(-1.2,0.7) | 0.579 | -0.6(-0.9,-0.3) | <0.001* | 1.0(0.6,1.4) | <0.001* |
| CI (L/min*m^2^) | 0.0(-0.4,0.5) | 0.867 | -0.3(-0.5,-0.2) | <0.001* | 0.6(0.3,0.8) | <0.001* |
| rScO_2_ (%) | -0.6(-6.9,5.7) | 0.858 | -0.0(-1.0,0.9) | 0.944 | 1.0(-0.4,2.5) | 0.167 |
| MCAvm (cm/s) | -1.4(-8.8,6.0) | 0.705 | 3.4(1.5,5.2) | <0.001* | 1.9(-0.8,4.7) | 0.173 |
| PI | 0.1(-0.1,0.3) | 0.170 | -0.0(-0.1,0.1) | 0.643 | -0.0(-0.1,0.1) | 0.930 |
| RI | 0.0(-0.0,0.1) | 0.159 | -0.0(-0.0,0.0) | 0.379 | 0.0(-0.0,0.0) | 0.590 |
| CO_X_ | 0.1(-0.2,0.3) | 0.620 | 0.13(-0.02,0.27) | 0.0841 | -0.2(-0.4,0.1) | 0.185 |
| M_Xa_ | 0.05(-0.14,0.25) | 0.585 | 0.10(-0.02,0.22) | 0.093 | 0.0(-0.2,0.2) | 0.959 |

**Note:** Data are shown as mean (SD) or median [quartile range]. ^*^p<0.05 between two groups. SBP: systolic blood pressure; MAP: Mean arterial blood pressure; DBP: diastolic blood pressure; HR: heart rate; SV: stroke volume; SVI: stroke volume index; CO: cardiac output; CI: cardiac index; rScO_2_: regional cerebral oxygen saturation; MCAvm: mean blood velocity of the middle cerebral artery_;_ PI: pulsatility index_;_ RI: resistance index; CO_X_: cerebral oximetry index; M_Xa_: mean flow index. time: before and after vasopressor. Using linear mixed model analysis
